# A3 Adenosine Receptor Agonists as Multisystem Disease Modifiers: From Molecular Signaling to Clinical Translation

**DOI:** 10.3390/biom16060907

**Published:** 2026-06-18

**Authors:** Pnina Fishman

**Affiliations:** Can-Fite BioPharma Ltd., Ramat Gan 5257346, Israel; pnina@canfite.co.il

**Keywords:** A3 adenosine receptor, piclidenoson, namodenoson, purinergic signaling, inflammation, cancer, fibrosis, metabolic dysfunction-associated steatohepatitis, neuroprotection, ischemic stroke, osteoarthritis, psoriasis, rheumatoid arthritis, Lowe syndrome, immunomodulation, disease-modifying therapy

## Abstract

The A3 adenosine receptor (A3AR) is a stress-inducible G-protein-coupled receptor that is selectively upregulated in inflamed, hypoxic, and fibrotic tissues as well as in many malignancies, while remaining weakly expressed in most normal organs. This distinctive expression pattern provides a strong biological basis for pathology-selective pharmacology. Activation of A3AR by highly selective agonists, including piclidenoson (IB-MECA) and namodenoson (Cl-IB-MECA), initiates signaling through Gi proteins and phospholipase C (PLC), which in turn regulate a coordinated network of downstream intracellular pathways, including PI3K/Akt, NF-κB, MAPKs, and Wnt/β-catenin, resulting in suppression of inflammation, inhibition of pathological cell survival, and protection of metabolically stressed tissues. Over the three decades, extensive preclinical studies have demonstrated that A3AR agonism exerts anti-cancer, anti-fibrotic, immunomodulatory, neuroprotective, and organ-protective effects across diverse disease models, including hepatocellular carcinoma, pancreatic cancer, psoriasis, osteoarthritis, metabolic dysfunction-associated steatohepatitis, ischemic stroke, neurodegeneration, ophthalmic disorders, and inherited metabolic syndromes. Importantly, these mechanistic insights have been translated into clinical programs, with piclidenoson and namodenoson demonstrating favorable safety profiles and disease-modifying activity in inflammatory, fibrotic, and oncologic indications. This review integrates molecular, cellular, and translational evidence to highlight A3AR activation as a unifying therapeutic principle for diseases driven by inflammation, oxidative stress, hypoxia, and dysregulated cell survival, positioning selective A3AR agonists as first-in-class agents targeting the A3AR, with broad clinical applicability across multiple disease domains.

## 1. Introduction

Adenosine is an evolutionarily conserved purine nucleoside that functions as a local metabolic and stress-response signal in virtually every tissue [1,2]. Adenosine is continuously generated under physiological conditions as part of normal cellular metabolism, primarily through intracellular hydrolysis of S-adenosylhomocysteine and the basal turnover of ATP, as well as extracellularly via ectonucleotidases (CD39 and CD73) that convert released ATP to adenosine. Under normoxic conditions, adenosine levels are tightly regulated by rapid phosphorylation via intracellular adenosine kinase and degradation by adenosine deaminase, maintaining low extracellular concentrations that contribute to tissue homeostasis. During hypoxia, ischemia, inflammation, or mechanical stress, ATP breakdown is markedly accelerated and adenosine clearance is reduced, leading to substantial extracellular accumulation and enhanced activation of four G-protein-coupled adenosine receptors: A1, A2A, A2B, and A3 [1,2,3]. These receptor subtypes differ in tissue distribution, G-protein coupling, and downstream second-messenger pathways, forming a tightly regulated system that fine-tunes cellular responses to metabolic and inflammatory stress [2,3]. Among the four adenosine receptor subtypes, A1 and A2A receptors have been extensively studied and are well-established modulators of neuronal activity, cardiovascular function, and inflammation. However, the A3AR (A3AR) has emerged as an especially attractive therapeutic target in the context of chronic inflammatory, oncologic, and neurodegenerative diseases, primarily due to its disease-selective overexpression and unique signaling profile. In contrast to A1 and A2A receptors, which are widely expressed in normal tissues and associated with dose-limiting cardiovascular and central nervous system effects, A3AR is expressed at low levels under physiological conditions but is markedly upregulated in pathological environments. This differential expression enables selective targeting of diseased tissues while minimizing systemic toxicity. Furthermore, A3AR activation exerts context-dependent effects, promoting apoptosis in malignant and inflammatory cells while conferring cytoprotection in normal or stressed tissues, a feature that distinguishes it from other adenosine receptor subtypes and supports its development as a disease-modifying therapeutic target [4,5].

A3AR was cloned in the early 1990s from rat and human tissues as the last member of the adenosine receptor family to be identified [6,7]. It couples predominantly to inhibitory Gi proteins, leading to suppression of adenylyl cyclase and a reduction in intracellular cyclic AMP (cAMP) [8]. In contrast to A1 and A2A receptors, A3AR is expressed at low levels in most normal tissues but is markedly upregulated in pathological environments, including inflamed tissues, hypoxic tumors, and fibrotic organs [5,9]. This disease-associated expression is driven in part by transcription factors such as NF-κB, AP-1, GATA-1, CREB and HIF-1α, which bind regulatory elements in the A3AR promoter under inflammatory and hypoxic conditions [5,10,11]. As a result, A3AR agonists can preferentially act in diseased tissues while largely sparing normal organs, a highly desirable property for chronic pharmacological intervention.

**Figure 1:**
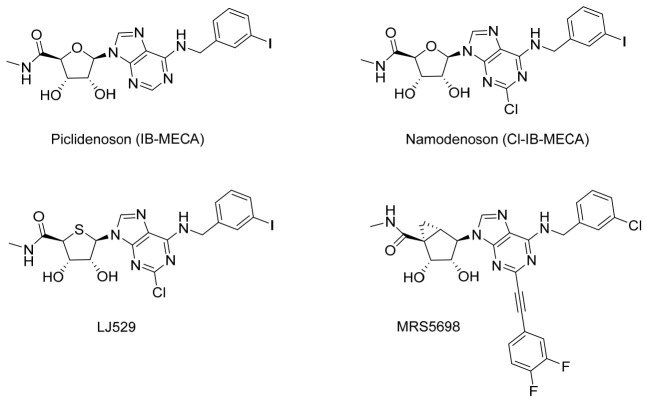
Chemical structures of representative A3 adenosine receptor (A3AR) agonists, including piclidenoson (IB-MECA), namodenoson (Cl-IB-MECA), LJ529, and MRS5698. These compounds share a nucleoside scaffold with structural modifications that confer high affinity and selectivity for A3AR and have been investigated across oncology, inflammatory, and neurodegenerative disease models.

A3AR is a seven-transmembrane G-protein-coupled receptor with a well-defined orthosteric binding pocket that accommodates adenosine analogs bearing halogen or alkyl substitutions at the N6 position [12,13,14]. Structure–activity studies pioneered by Jacobson and colleagues led to the development of highly selective A3AR agonists, including IB-MECA, Cl-IB-MECA, LJ529 and MRS5698 [12,13,15]; Figure 1.

These compounds were subsequently in-licensed by Can-Fite BioPharma and developed under the names piclidenoson (IB-MECA) and namodenoson (Cl-IB-MECA), respectively. These molecules display nanomolar affinity and high selectivity for A3AR over A1 and A2A receptors [12,15].

Crystallographic and molecular-modeling studies have shown that subtle conformational features of the A3AR, particularly within the third intracellular loop and C-terminal tail, facilitate preferential coupling to Gi/Go proteins and activation of multiple downstream signaling pathways, which are described in detail in the following section [16,17,18]. Through these pathways, A3AR regulates a broad spectrum of cellular functions, including inhibition of inflammatory cytokine production, induction of apoptosis in malignant cells, attenuation of oxidative stress, and protection from ischemic injury [4,5,19,20,21]. In the nervous system, A3AR signaling limits microglial activation and oxidative damage, contributing to neuroprotection [22,23,24].

Over the past two decades, selective A3AR agonists have progressed from preclinical validation to late-stage clinical evaluation. Piclidenoson and namodenoson represent first-in-class orally bioavailable agents that exploit the disease-selective biology of this receptor [25,26,27]. In parallel, numerous academic groups have established A3AR as a mediator of tissue protection in cardiovascular, neurological, renal, and ophthalmic disease models [22,23,24,27,28,29]. Collectively, these studies extend the relevance of A3AR from oncology and autoimmune disorders to fibrosis, ischemia–reperfusion injury, neuropathic pain, vascular dementia, and Lowe syndrome.

This review synthesizes the expanding mechanistic and translational evidence base surrounding A3AR pharmacology, focusing on preclinical studies that elucidate receptor signaling and therapeutic potential across multiple organ systems, together with an overview of ongoing clinical development. By integrating molecular, pharmacologic, and clinical perspectives, this review highlights A3AR activation as a unifying therapeutic principle for diseases driven by inflammation, hypoxia, oxidative stress, and dysregulated cell survival.

### 1.1. Molecular and Cellular Mechanisms of A3AR Signaling

Ligand binding to A3AR initiates coupling to Gi/Go proteins, resulting in inhibition of adenylyl cyclase and a consequent decrease in intracellular cAMP levels. Reduced cAMP suppresses protein kinase A (PKA) activity, thereby altering the phosphorylation status of multiple transcriptional regulators and signaling intermediates [30,31,32]. This primary signaling event diverges into several intracellular cascades that account for the pleiotropic biological actions of A3AR.

In parallel with Gi-mediated signaling, A3AR activation recruits phospholipase C-β (PLCβ) via Gβγ subunits [31,32]. Activated PLCβ hydrolyzes phosphatidylinositol-4,5-bisphosphate (PI(4,5)P_2_) to generate inositol-1,4,5-trisphosphate (IP_3_) and diacylglycerol (DAG), leading to intracellular Ca^2+^ release and activation of protein kinase C (PKC) isoforms, respectively [33,34,35]. The combined reduction in cAMP and increase in Ca^2+^/PKC signaling creates a distinct intracellular fingerprint that differentiates A3AR from other adenosine receptor subtypes [31,34]. A3AR signaling intersects with multiple canonical kinase networks. In tumor and immune cells, A3AR agonists suppress the PI3K/Akt/mTOR pathway, resulting in downregulation of pro-survival and proliferative signals [12,17,36]. Reduced Akt phosphorylation leads to inhibition of mTOR, S6 kinase, and GSK-3β, thereby shifting the cellular balance toward apoptosis and growth arrest [12,36]. In parallel, A3AR activation inhibits NF-κB signaling by stabilizing the inhibitory protein IκB and preventing its degradation, leading to reduced NF-κB nuclear translocation and transcriptional activity [34,35,36,37,38]. This results in decreased expression of inflammatory mediators, including TNF-α, IL-1β, IL-6, IL-8, IL-23, and IL-17 [19,20,21].

A3AR also modulates the Wnt/β-catenin pathway, a mechanism of particular relevance in oncology, inflammation and fibrosis. A3AR agonists downregulate β-catenin expression and inhibit its nuclear translocation, thereby reducing transcription of genes that promote proliferation. In hepatocellular and pancreatic tumors, inhibition of the Wnt/β-catenin signaling pathway sensitizes malignant cells to apoptosis [39,40,41].

In Chinese Hamster Ovary (CHO) cells transfected with the human adenosine A3 receptor and in N13 microglia cells which endogenously express A3 receptors, A3AR activation modulates mitogen-activated protein kinases (MAPKs), including ERK1/2, JNK, and p38, which regulate stress responses, cytokine production, and neuronal survival [41].

Beyond cytosolic signaling, A3AR exerts profound effects on mitochondrial function. In cancer cells and activated fibroblasts, A3AR activation promotes expression of the pro-apoptotic protein Bax while downregulating anti-apoptotic Bcl-2, leading to cytochrome-c release and caspase activation [25,41]. Conversely, in non-malignant metabolically stressed tissues such as myocardium and brain, A3AR stimulation stabilizes mitochondrial membrane potential, reduces reactive oxygen species (ROS) generation, and preserves ATP synthesis, thereby conferring cytoprotection [34,42]. These divergent effects are explained by differential receptor density and cellular context, with A3AR activation selectively inducing apoptosis in overexpressing pathological cells while protecting healthy or hypoxic tissues [4,42].

Like many G-protein-coupled receptors, A3AR undergoes phosphorylation by GPCR kinases (GRKs) and by β-arrestin recruitment, internalization and desensitization [43]. This is followed by receptor resensitization and with prolonged (up to 24 h) agonist exposure, contributing to sustained receptor signaling during chronic disease [44,45].

Collectively, A3AR signaling constitutes a multi-axis regulatory network comprising a membranal arm (Gi–cAMP–PKA), an inflammatory arm (NF-κB and inflammatory cytokine inhibition), a growth-control arm (PI3K/Akt/mTOR and Wnt/β-catenin suppression), and a cytoprotective arm (mitochondrial stabilization and antioxidant defense). Through these integrative mechanisms, A3AR activation restores cellular homeostasis in tissues exposed to metabolic and inflammatory stress, providing a molecular framework for its broad efficacy across models of cancer, inflammatory disease, fibrosis, neurodegeneration, and organ ischemia.

The principal signaling axes and integrated biological effects of A3AR activation are summarized in Table 1.

**Figure 2:**
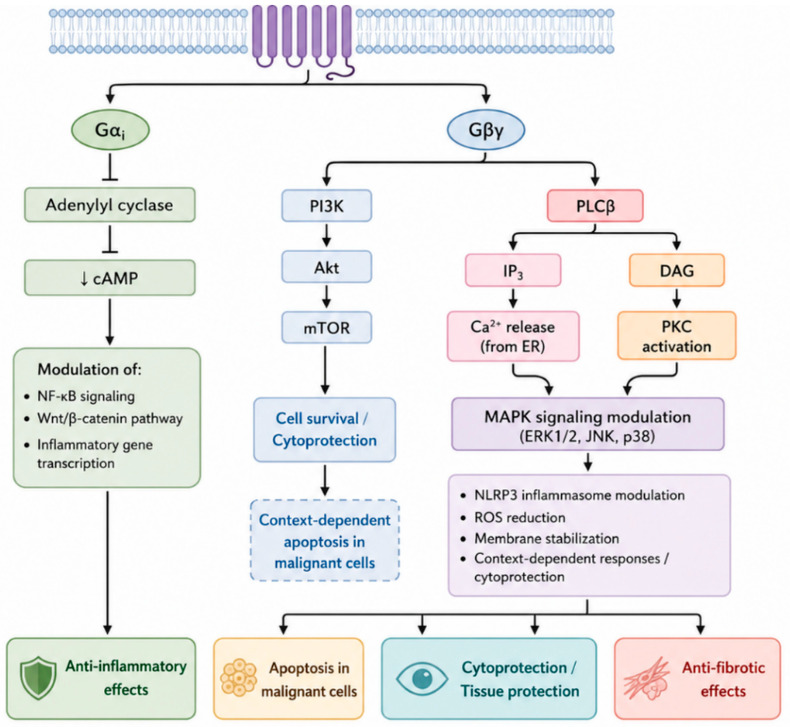
Major signaling axes and integrated biological effects of A3 adenosine receptor (A3AR) activation. Binding of adenosine or selective agonists to A3AR primarily engages Gi proteins and Gβγ-mediated phospholipase C (PLC) signaling, resulting in inhibition of adenylyl cyclase, reduced cAMP levels, and activation of PLCβ-dependent second messengers. These proximal events regulate downstream pathways including PI3K/Akt/mTOR, NF-κB, Wnt/β-catenin, MAPKs (ERK, JNK, p38), the NLRP3 inflammasome, and mitochondrial stress responses. Collectively, these signaling networks mediate anti-inflammatory, anti-proliferative, anti-fibrotic, neuroprotective, and context-dependent cytotoxic or cytoprotective effects.

The major signaling architecture of A3AR activation is schematically summarized in Figure 2.

### 1.2. Preclinical Pharmacology and Disease Applications

Recent comprehensive reviews have summarized the expanding biology and therapeutic potential of A3AR agonists across oncology, inflammation, and organ-protective indications, highlighting their unique disease-selective pharmacology and broad translational relevance.

Representative preclinical disease models and mechanistic outcomes across organ systems are summarized in Table 2.

## 2. Anti-Cancer Activity

A3AR agonists exert anti-cancer activity across multiple malignancies through a dual mechanism that combines direct tumor cell cytotoxicity with modulation of the tumor microenvironment (TME) [41,45]. The most advanced body of evidence exists in hepatocellular carcinoma (HCC), with expanding preclinical and translational data in pancreatic ductal adenocarcinoma (PDAC) and at the same time, cytoprotective effects towards normal tissues [39,40,51].

In HCC, A3AR is frequently overexpressed in tumor tissue compared with adjacent normal liver, enabling selective pharmacologic targeting. Activation of A3AR suppresses tumor cell proliferation and induces apoptosis through inhibition of PI3K/Akt and NF-κB signaling, together with downregulation of Wnt/β-catenin-dependent transcription. These coordinated effects impair cell-cycle progression and reduce survival signaling, resulting in marked inhibition of tumor growth in xenograft and orthotopic HCC models [39].

In PDAC, A3AR agonists inhibit tumor growth in vitro and in vivo at nanomolar concentrations in an A3AR-dependent manner, promoting apoptosis and suppressing pro-survival pathways including PI3K/Akt, NF-κB, RAS, and Wnt/β-catenin signaling [40]. Importantly, a combination of A3AR agonists with gemcitabine or other cytotoxic agents produces additive or synergistic anti-tumor activity in both PDAC and HCC, supporting their use in combination regimens for these highly aggressive malignancies [65].

Beyond the liver and pancreas, anti-tumor effects of A3AR agonists have been demonstrated in experimental models of melanoma, colorectal carcinoma, breast cancer, and prostate cancer, indicating a broad anti-neoplastic profile driven by receptor overexpression in diverse tumor types [66,67,68].

Notably, the role of A3AR in tumor biology appears to be context-dependent and influenced by the tumor microenvironment. While the majority of studies support an anti-tumor effect of A3AR activation, earlier reports and selected experimental systems have suggested that A3AR signaling may, under certain conditions, support tumor cell survival or proliferation [52]. These seemingly divergent findings likely reflect differences in receptor expression levels, ligand concentration, and cellular context, highlighting the complexity of A3AR-mediated signaling in cancer. Accordingly, the regulatory role of A3AR overexpression in some tumor types remains incompletely understood and warrants further investigation.

A particularly important interaction exists between A3AR signaling and immune checkpoint regulation. Aberrant Wnt/β-catenin activation is a major driver of resistance to PD-1/PD-L1 immune checkpoint inhibitors (ICIs) in tumor cells [69,70,71]. A3AR agonists downregulate β-catenin and are therefore expected to reduce PD-L1 expression on tumor cells, potentially reversing immune exclusion and enhancing susceptibility to immune-mediated killing. Consistent with this mechanistic framework, exploratory in vitro studies have evaluated the effect of namodenoson in combination with PD-L1-targeting strategies on tumor immune signaling. In Hep3B hepatocellular carcinoma cells, namodenoson (10 nM, 48 h incubation) was associated with a reduction in PD-L1 protein expression (~51%), accompanied by β-catenin downregulation and inhibition of cell proliferation (~65%), supporting a potential role in modulating tumor immune evasion pathways.

Clinical development of namodenoson began with a Phase I study in healthy volunteers, followed by a Phase I/II study in HCC (NCT00790218) and a Phase II study in Child–Pugh B cirrhosis patients with advanced HCC (NCT02128958) [72,73]. A pivotal Phase III trial in this high-risk population is currently ongoing (NCT05201404) under FDA and EMA guidance. In PDAC, a Phase IIa study of namodenoson monotherapy is ongoing, with all patients enrolled, and combination studies with chemotherapy and ICIs under consideration. Across all clinical studies, namodenoson has demonstrated a placebo-like safety profile [74]. The compound received FDA Fast Track designation in 2015 and Orphan Drug designation for HCC in 2012, underscoring its potential to address a major unmet medical need.

The current clinical development landscape of A3AR agonists is summarized in Table 3.

## 3. Inflammation and Autoimmune Disorders

A3AR agonists exert potent anti-inflammatory activity by suppressing key cytokines that drive chronic immune-mediated diseases, including TNF-α, IL-6, IL-8, MIP-1α, IL-17, IL-23, and matrix metalloproteinases (MMPs). These effects are mediated primarily through inhibition of NF-κB signaling and stabilization of IκB [80,81,82,83,84].

### 3.1. Osteoarthritis

Shkhyan and colleagues first demonstrated that A3AR plays a protective role in cartilage homeostasis. Genetic deletion of A3AR in mice leads to spontaneous cartilage degeneration and osteoarthritis-like structural changes, providing strong evidence that basal A3AR activity protects against joint destruction [53]. Mechanistic analyses demonstrated that loss of A3 adenosine receptor (A3AR) signaling promotes chondrocyte hypertrophy and extracellular matrix degradation through activation of CaMKII and RUNX2 pathways, both strongly implicated in osteoarthritis progression, while A3AR activation suppresses these signaling axes and preserves cartilage integrity, consistent with the broader role of GPCR-mediated signaling in bone and cartilage homeostasis [54,55].

Subsequent studies demonstrated that A3AR activation suppresses NF-κB and PI3K/Akt signaling in inflammatory cells and chondrocytes, reducing production of catabolic enzymes and inflammatory mediators. Treatment with piclidenoson significantly attenuated cartilage destruction and improved pain-related behaviors in rodent OA models [56]. More recently, A3AR agonism was shown to suppress activation of the NLRP3 inflammasome and downstream caspase-1/GSDMD-mediated pyroptosis, linking purinergic signaling to emerging mechanisms of inflammatory cell death in osteoarthritic cartilage [47,48].

Veterinary clinical data further support translational relevance. In a multicenter trial in dogs with naturally occurring osteoarthritis, the A3AR agonist piclidenoson significantly improved Liverpool Osteoarthritis in Dogs (LOAD) and Visual Analog Scale (VAS) pain scores in a dose- and time-dependent manner, consistent with disease-modifying activity [57].

### 3.2. Rheumatoid Arthritis

Rheumatoid arthritis (RA) is characterized by synovial hyperplasia, immune-cell infiltration, and progressive joint destruction driven by pro-inflammatory cytokines and dysregulated intracellular signaling. A3AR is selectively overexpressed in peripheral blood mononuclear cells and synovial tissue of RA patients, making it an attractive therapeutic target [5,58]. Activation of A3AR suppresses PI3K/Akt and NF-κB signaling, resulting in reduced production of TNF-α, IL-6, IL-1β, IL-8, IL-17 and IL-23 [56,81].

In animal models of inflammatory arthritis, oral piclidenoson significantly reduced clinical arthritis scores, joint swelling, cartilage damage, and bone erosion, with efficacy comparable to standard disease-modifying antirheumatic drugs (DMARDs) and without evidence of global immunosuppression [59].

Clinical studies confirmed an excellent safety profile. A Phase 2a monotherapy study demonstrated significant ACR20/50/70 responses, with optimal efficacy observed at an oral dose of 1 mg administered twice daily [85]. Phase 2b combination studies with methotrexate (MTX) failed, likely due to MTX-induced downregulation of A3AR expression [74]. In contrast, a placebo-controlled Phase 2b monotherapy study met its primary endpoint, with particularly strong responses in treatment-naïve patients [86]. A Phase 3 trial confirmed safety and superiority over placebo but did not achieve non-inferiority to MTX, leading to discontinuation of RA development while validating A3AR biology in human disease [87].

### 3.3. Psoriasis

Psoriasis is driven by dysregulated interactions between keratinocytes and immune cells, particularly Th17 and Th1 lymphocytes, leading to overproduction of TNF-α, IL-17, IL-23, and IL-22. A3AR is selectively overexpressed in PBMCs and lesional skin of psoriasis patients, supporting its relevance as a biomarker-linked therapeutic target [5].

In vitro studies demonstrated that piclidenoson directly inhibits keratinocyte proliferation and suppresses NF-κB and PI3K/Akt signaling in human keratinocytes, confirming receptor-specific anti-inflammatory and anti-proliferative activity [80]. In vivo, selective A3AR agonists (MRS5698 and photocaged MRS7344) suppressed IL-23-driven psoriasiform inflammation in mouse models, reducing epidermal hyperplasia and immune-cell infiltration [60].

Can-Fite also demonstrated efficacy of piclidenoson in the imiquimod-induced psoriasis model, showing significant reductions in clinical skin scores demonstrating the topical effect of the drug (unpublished data). These findings translated clinically: Phase 2 and Phase 2/3 trials demonstrated safety and dose-dependent PASI improvements [88,89], and the Phase 3 COMFORT-1 trial showed statistically significant PASI-75 responses and superior tolerability compared with apremilast [90]. The COMFORT-2 Phase 3 study is currently enrolling patients.

## 4. Metabolic Dysfunction-Associated Steatohepatitis (MASH)

MASH, formerly non-alcoholic steatohepatitis (NASH), is characterized by hepatic steatosis, inflammation, hepatocyte injury, and progressive fibrosis driven by metabolic stress and innate immune activation.

In several rodent models including diet- and toxin-induced steatohepatitis, oral administration of the selective A3AR agonist namodenoson significantly reduced hepatic steatosis, lobular inflammation, hepatocyte ballooning, and fibrosis. These histological improvements were associated with downregulation of pro-inflammatory and pro-fibrotic mediators, including TNF-α and α-smooth muscle actin (α-SMA), reflecting inhibition of hepatic stellate cell activation and upregulation of adiponectin [61].

At the molecular level, A3AR agonists inhibit PI3K/Akt and NF-κB signaling in hepatocytes, Kupffer cells, and stellate cells, leading to suppression of inflammatory cytokine production and induction of apoptosis in activated fibrogenic cells. This integrated immune–metabolic and anti-fibrotic mechanism distinguishes A3AR agonism from therapies that target only lipid metabolism or inflammation.

Clinical translation supports these findings. In a Phase 2 study in MASH patients, namodenoson showed a very favorable safety profile. Evidence of liver-targeted biological activity included the following parameters: improvements in ALT and AST levels which decreased consistently during the study in a dose-dependent manner, an increase in serum adiponectin, a significant decrease in liver fat volume and a significant decrease in Fib-4 score, suggesting an inhibitory effect of the drug on fibrosis progression [75]. These results support A3AR agonism as a first-in-class, orally administered, disease-modifying approach that integrates anti-inflammatory, metabolic, and anti-fibrotic pathways. A Phase 2b study in MASH patients with biopsy-proven disease is currently ongoing.

## 5. A3AR Agonists in Neuroinflammatory and Neurodegenerative Diseases

A3AR is functionally expressed in microglia (a major cellular target), astrocytes, endothelial cells of the blood–brain barrier, and injured neurons, and its activation orchestrates neuroprotection through combined immunomodulatory and neuron-intrinsic survival mechanisms, including regulation of glial metabolism and inflammatory signaling. Mechanistically, A3AR agonism suppresses neuroinflammatory transcriptional programs (notably NF-κB-driven cytokine networks), reduces inducible nitric oxide synthase (iNOS) and redox stress signaling, and rebalances kinase pathways such as ERK/JNK/p38 MAPKs in a context-dependent manner. In parallel, A3AR activation stabilizes mitochondrial function and limits oxidative damage, including suppression of NADPH oxidase-dependent ROS generation, thereby preserving neuronal integrity in settings of hypoxia–ischemia and inflammatory injury. Transcriptomic and mechanistic studies further support the concept that A3AR agonists can shift activated microglia away from damaging inflammatory phenotypes, providing a rational foundation for disease modification in disorders where microglial activation drives progression [76,77,78,91].

### 5.1. Neuropathic Pain

A compelling body of work (particularly from Salvemini’s group and collaborators) supports A3AR agonists as non-opioid analgesics that target neuroinflammation and oxidative stress in the spinal cord. In a seminal paclitaxel-induced neuropathic pain model, the selective A3AR agonist IB-MECA (piclidenoson) prevented development of neuropathic pain by inhibiting spinal NADPH oxidase activation and downstream redox-dependent signaling, consistent with disease-relevant suppression of glial-driven sensitization [63]. Complementary medicinal-chemistry and preclinical studies identified highly selective A3AR agonists such as MRS5698, a highly selective A3AR agonist developed by academic groups, which has demonstrated robust anti-allodynic activity in chronic neuropathic pain models, strengthening the translational case for this target class [50,63,92]. More recently, mechanistic work has extended these findings by implicating mitochondrial A3AR-dependent pathways in chemotherapy-induced neuropathy and by confirming that A3AR agonists can prevent pain development without opioid-like tolerance or reward behavior. Additional studies show that A3AR agonism can act as a distinct analgesic strategy that may complement or replace opioid approaches in selected neuropathic pain contexts [46,49,64,92,93,94].

### 5.2. Ischemic Stroke and Brain Ischemia

Preclinical studies demonstrate that A3AR agonists can reduce ischemic brain injury in models of focal and global cerebral ischemia. Early work showed that A3AR activation reduces infarct burden and mitigates downstream inflammatory and apoptotic cascades in rodent cerebral ischemia. In a well-cited mechanistic study, the selective A3AR agonist LJ529 reduced brain ischemic injury through anti-inflammatory and cytoprotective mechanisms consistent with stabilization of cellular stress responses and limitation of cell death pathways. Additional experimental data support that sustained A3AR agonism can preserve ischemia-sensitive neuronal markers (e.g., MAP-2), reduce nitric oxide synthase induction, and modulate glial reactivity following ischemic insults, aligning with both anti-inflammatory and neuroprotective effects [62,95,96].

### 5.3. Alzheimer’s Disease and Neuroinflammation

While A3AR agonists remain less clinically developed in Alzheimer’s disease than A2A receptor antagonists, accumulating evidence supports a role for A3AR signaling in amyloidogenic processing and microglial phenotype control. In cultured neurons, caffeine was shown to suppress amyloid-β protein precursor (AβPP) internalization and reduce amyloid-β generation at least partly via A3AR-mediated mechanisms, providing a mechanistic link between A3AR signaling and amyloidogenic processing [97]. Additional study on A3AR signaling in Alzheimer’s disease highlights that A3AR can influence microglial activation states and inflammatory outputs, supporting continued exploration of A3AR-directed strategies in amyloid-associated neuroinflammation [97].

### 5.4. Vascular Dementia and Chronic Hypoperfusion

A major advance in the field emerged from work integrating mouse vascular dementia (VaD) transcriptomics with human single-nucleus RNA sequencing, which identified disruption of an intercellular CD39–A3AR signaling system in the neurovascular niche. In these studies, pharmacologic restoration of CD39–A3AR signaling using an A3AR-specific agonist (noted as clinically relevant) improved tissue integrity and behavioral outcomes in a VaD model, supporting A3AR activation as a potential repair-promoting pathway in chronic hypoperfusion-linked cognitive impairment [63,98]. These findings align with the broader concept that A3AR agonists can support neurovascular repair by dampening damaging microglial activation while promoting recovery programs in the injured white matter environment as described above.

### 5.5. Cardiovascular Protection

A3ARs are expressed in cardiomyocytes and vascular endothelial cells, and are upregulated by ischemic and hypoxic stress, contributing to endogenous cardioprotective signaling, reducing infarct size and enhancing cellular survival in ischemia–reperfusion models. [99]. In experimental myocardial ischemia–reperfusion injury, A3AR activation limits infarct size, preserves left-ventricular function and improves post-ischemic recovery [50]. Namodenoson and other A3AR agonists stabilize mitochondrial membrane potential, inhibit cytochrome-c release, and prevent caspase-3-mediated cardiomyocyte apoptosis, thereby preserving ATP generation and contractile function [100,101,102,103].

In parallel, A3AR activation suppresses NADPH-oxidase-dependent ROS generation and reduces inflammatory leukocyte adhesion to ischemic endothelium, limiting microvascular obstruction and reperfusion injury. Beyond ischemia–reperfusion injury, A3AR activation has been shown to attenuate doxorubicin-induced cardiomyocyte injury by reducing mitochondrial dysfunction and apoptotic signaling, supporting its potential role as a cardioprotective adjuvant during chemotherapy [103,104].

## 6. Ophthalmology and Retinal Protection

A3AR is expressed in retinal pigment epithelial cells, retinal ganglion cells, and microglia, and its expression is increased under conditions of ocular inflammation and ischemic stress. Activation of adenosine A3 receptor protects retinal ganglion cells from degeneration induced by ocular hypertension [30,96]. In models of uveitis and glaucoma, A3AR agonists suppress NF-κB-dependent inflammatory cytokines, inhibit leukocyte infiltration, and preserve retinal ganglion-cell survival [105,106].

Although oral piclidenoson showed limited efficacy in late-stage glaucoma trials, the strong preclinical evidence for direct retinal A3AR-mediated neuroprotection supports continued development via topical ocular formulations that bypass systemic dilution and maximize retinal exposure [107].

## 7. Lowe Syndrome (OCRL Deficiency)

Lowe syndrome is an X-linked multisystem disorder caused by mutations in the OCRL gene encoding phosphatidylinositol-5-phosphatase, leading to abnormal PI(4,5)P_2_ accumulation, defective endosomal trafficking, and cytoskeletal instability in kidney, brain, and eye tissues [108]. A seminal discovery by the TIGEM–Federico II Naples group demonstrated that A3AR activation corrects these defects at their biochemical root. In OCRL-deficient cells and mouse models, piclidenoson normalized PI(4,5)P_2_ levels through A3AR-PLC-dependent hydrolysis, restored endosomal morphology, reduced inflammatory signaling, and improved renal tubular function, including reduced proteinuria and improved reabsorption capacity. This unique mechanism represents a true disease-modifying rescue, rather than symptomatic management. These findings led to a translational partnership between Fondazione Telethon and Can-Fite BioPharma to advance piclidenoson into a Phase 2 clinical study at Bambino Gesù Children’s Hospital in Rome. This marks the first application of A3AR modulation in a rare inherited metabolic disease. A patent has been filed as of the use of A3AR agonist as a treatment for Lowe syndrome (WO 2025/011999 A1).

## 8. Safety Profile of Piclidenoson and Namodenoson

Across all clinical studies, namodenoson and piclidenoson have demonstrated a favorable safety profile, with most adverse events being mild. Reported side effects have included headache, gastrointestinal discomfort, and fatigue, and overlapped with those of placebo-treated patients. Importantly, no signal of immunosuppression or organ-specific toxicity has been consistently observed across studies. This safety profile is consistent with the relatively low expression of A3AR in normal tissues and its selective upregulation in pathological conditions. Nevertheless, as with other G-protein-coupled receptor-targeted therapies, long-term effects and potential context-dependent responses warrant continued evaluation in larger clinical trials [74].

## 9. Challenges and Limitations of A3AR-Targeted Therapies

Despite the largely consistent therapeutic profile of A3AR agonists, some earlier and seemingly contradictory findings have been reported in the literature. It should be noted that earlier studies explored A3AR antagonists as potential therapeutic agents, particularly in asthma and certain cancer models, based on observations in rodent systems where A3AR activation could promote bronchoconstriction or modulate hypoxia-related pathways. In addition, A3AR antagonists have been reported to reduce intraocular pressure, whereas A3AR agonists demonstrate neuroprotective effects in retinal tissues. These seemingly contradictory findings likely reflect species differences, tissue-specific receptor expression, and context-dependent signaling mechanisms. Current evidence in human systems and clinical studies predominantly supports a protective and anti-inflammatory role of A3AR activation. Like other G-protein-coupled receptors, A3AR is subject to desensitization, internalization, and context-dependent signaling, which may influence long-term efficacy in chronic indications. In addition, variability in receptor expression across patient populations may contribute to heterogeneous clinical responses and may partly explain why some clinical trials did not meet primary endpoints compared to standard-of-care therapies. Consistent with this, despite more than two decades of clinical development, A3AR agonists have not yet achieved regulatory approval, reflecting the challenges of translating receptor biology into consistent clinical benefit across heterogeneous patient populations.

Furthermore, while A3AR is generally overexpressed in pathological tissues, its role in tumor biology is complex and may be influenced by the tumor microenvironment. Some studies have suggested that under certain conditions, A3AR signaling may support tumor survival or proliferation, highlighting the need for a more nuanced understanding of receptor function in different cellular contexts.

From a clinical development perspective, the use of A3AR agonists may require careful patient stratification based on receptor expression levels, as well as optimization of combination strategies with established therapies such as chemotherapy or immune checkpoint inhibitors.

Finally, although a substantial body of preclinical and clinical data supports the therapeutic potential of A3AR agonists, further independent studies and large-scale clinical trials are needed to fully establish their efficacy across diverse disease indications.

## 10. Conclusions

Taken together, the accumulated body of evidence reviewed here clearly establishes the A3AR as a central regulator of tissue responses to metabolic, inflammatory, and hypoxic stress. Unlike other adenosine receptor subtypes, A3AR displays a disease-inducible expression profile, being markedly upregulated in malignant, inflamed, ischemic, and fibrotic tissues while remaining minimally expressed in most healthy organs. This feature creates a pharmacologic window that enables selective targeting of pathological cells with minimal off-target toxicity, a property that is uncommon among G-protein-coupled receptor targets and highly advantageous for chronic systemic therapy.

At the mechanistic level, A3AR activation integrates several interconnected signaling axes that converge on restoration of cellular homeostasis. Suppression of the PI3K/Akt/mTOR and Wnt/β-catenin pathways limits aberrant proliferation, fibrosis, and immune evasion, while inhibition of NF-κB and STAT3 reduces pro-inflammatory cytokine networks that sustain chronic disease. At the same time, A3AR signaling stabilizes mitochondrial function, reduces oxidative stress, and preserves bioenergetic capacity in neurons, cardiomyocytes, renal tubular cells, and endothelial cells exposed to ischemic or metabolic injury. The ability of a single receptor system to simultaneously regulate inflammation, cell survival, fibrosis, and tissue repair provides a coherent explanation for the remarkably broad therapeutic profile of A3AR agonists across oncology, immunology, neurology, cardiology, hepatology, and rare genetic diseases.

Importantly, these mechanistic principles have translated into consistent in vivo efficacy. In cancer models, A3AR agonists directly induce tumor cell apoptosis while reprogramming the tumor microenvironment toward immune permissiveness, including suppression of PD-L1 expression and β-catenin signaling. In autoimmune and inflammatory diseases such as rheumatoid arthritis, psoriasis, and osteoarthritis, A3AR agonism attenuates cytokine-driven tissue destruction without causing global immunosuppression. In metabolic and fibrotic liver disease, A3AR activation integrates anti-inflammatory, metabolic, and anti-fibrotic pathways, leading to histological improvement in experimental MASH. In the nervous system, the receptor functions as a master regulator of neuroinflammation, oxidative injury, and neuronal survival, conferring protection in stroke, neuropathic pain, Alzheimer’s disease, and vascular dementia. The extension of A3AR biology into rare inherited disorders such as Lowe syndrome further illustrates the receptor’s role in regulating phosphoinositide homeostasis and endosomal function, expanding its relevance beyond classical inflammation and cancer.

Clinical experience accumulated with piclidenoson and namodenoson confirms that these mechanistic advantages translate into an excellent safety profile, even with long-term oral administration. Across multiple indications, A3AR agonists have shown a placebo-like tolerability profile, consistent with their selective activity in diseased tissues. Moreover, emerging data indicate that baseline A3AR expression serves as a predictive biomarker for therapeutic response, providing a rational framework for patient stratification and precision medicine. This biomarker-driven approach may explain variable responses observed in earlier clinical trials and offers a path toward maximizing efficacy in future studies.

Taken together, the data support A3AR activation as a unifying, disease-modifying therapeutic strategy rather than a symptomatic intervention. By targeting the convergent molecular pathways that drive inflammation, oxidative stress, fibrosis, and pathological cell survival, A3AR agonists offer a platform capable of addressing complex, multi-factorial diseases that remain poorly served by current therapies. Continued integration of mechanistic insight, biomarker-guided clinical development, and rational combination strategies, particularly with immunotherapies and metabolic modulators, positions A3AR agonism as a powerful and versatile paradigm in modern translational medicine.

## Figures and Tables

**Table 1:** Major A3AR signaling axes and integrated biological effects.

Signaling Axis	Primary Molecular Components	Downstream Cellular Effects	Disease Relevance	References
Gi–cAMP pathway	↓ Adenylyl cyclase → ↓ cAMP → ↓ PKA	Modulation of transcriptional regulators and stress-response genes	Broad anti-inflammatory regulation	[5,25]
PLC–IP3/DAG–Ca^2+^ pathway	Gβγ → PLCβ activation → IP3 + DAG	Ca^2+^ mobilization; PKC activation	Immune modulation; neuronal signaling	[33,46]
PI3K/Akt/mTOR pathway	↓ Akt phosphorylation → ↓ mTOR/S6K	Reduced proliferation; apoptosis induction; growth arrest	Cancer; fibrosis; hyperproliferative disorders	[12,18]
NF-κB pathway	IκB stabilization → ↓ NF-κB nuclear translocation	↓ TNF-α, IL-1β, IL-6, IL-17, IL-23	RA; psoriasis; MASH; neuroinflammation	[19,20,21]
Wnt/β-catenin pathway	↓ β-catenin stabilization and nuclear entry	↓ PD-L1 expression; ↓ tumor survival signaling	HCC; PDAC; immune checkpoint resistance	[39,40]
NLRP3 inflammasome	↓ Caspase-1 activation; ↓ GSDMD cleavage	Reduced pyroptosis and inflammatory cell death	Osteoarthritis	[47,48]
Mitochondrial regulation	↑ Membrane potential stability; ↓ ROS; ↓ cytochrome-c release	Cytoprotection in stressed tissues; apoptosis in malignant cells	Stroke; cardioprotection; oncology	[49,50]

**Table 2:** Preclinical disease models and mechanistic outcomes of A3AR agonists.

Indication	Experimental Model	A3AR Agonist	Key Mechanisms Observed	Principal Outcome	References
Hepatocellular carcinoma	Xenograft/orthotopic models	Namodenoson	↓ PI3K/Akt; ↓ NF-κB; ↓ β-catenin	Tumor growth inhibition; apoptosis induction	[39,51]
Pancreatic ductal adenocarcinoma	In vitro + in vivo	Namodenoson	↓ RAS; ↓ Akt; ↓ Wnt signaling	Reduced proliferation; chemo sensitization	[40,52]
Osteoarthritis	A3AR knockout mice; OA models	Piclidenoson	↓ NF-κB; ↓ NLRP3; ↓ CaMKII/RUNX2	Reduced cartilage degeneration	[47,48,53,54,55,56,57]
Rheumatoid arthritis	Collagen-induced arthritis	Piclidenoson	↓ TNF-α; ↓ IL-6; ↓ IL-1β	Reduced arthritis scores and joint damage	[58,59]
Psoriasis	Imiquimod-induced model	Piclidenoson	↓ IL-23/Th17 axis	Reduced epidermal hyperplasia and inflammation	[60]
MASH	Diet- and toxin-induced models	Namodenoson	↑ Adiponectin; ↓ α-SMA; ↓ TNF-α	Reduced steatosis, inflammation, and fibrosis	[61]
Ischemic stroke	MCAO rodent models	LJ529/IB-MECA	↓ iNOS; ↓ ROS; ↓ microglial activation	Reduced infarct size; preserved neuronal markers	[62,63]
Neuropathic pain	Chemo-induced neuropathy	IB-MECA; MRS5698	↓ NADPH oxidase; ↓ glial activation	Prevention of mechanical allodynia	[49,64]

**Table 3:** Clinical development landscape of A3AR agonists.

Compound	Indication	Clinical Phase	Status	Key Findings	References
Piclidenoson	Rheumatoid arthritis	Phase 2–3	Completed	Excellent safety; validated biological activity	[61,75,76]
Piclidenoson	Psoriasis (COMFORT studies)	Phase 3	Ongoing	Significant PASI-75 responses; superior tolerability vs apremilast	[77,78,79]
Namodenoson	HCC (Child–Pugh B)	Phase 3	Ongoing	Orphan Drug and Fast Track designation; placebo-like safety	[53,80,81]
Namodenoson	Pancreatic cancer	Phase 2a	Fully enrolled	Monotherapy clinical evaluation	
Namodenoson	MASH	Phase 2b	Ongoing	↓ ALT/AST; ↑ adiponectin; ↓ Fib-4	[46]

## Data Availability

No new data were created or analyzed in this study. Data sharing is not applicable to this article.

## Abbreviations

A3AR, A3 adenosine receptor; AC, adenylyl cyclase; ADA, adenosine deaminase; Akt, protein kinase B; ATP, adenosine triphosphate; cAMP, cyclic adenosine monophosphate; DAG, diacylglycerol; ERK, extracellular signal-regulated kinase; GPCR, G-protein-coupled receptor; HCC, hepatocellular carcinoma; HIF-1α, hypoxia-inducible factor 1 alpha; IP3, inositol 1,4,5-trisphosphate; JNK, c-Jun N-terminal kinase; MAPKs, mitogen-activated protein kinases; mTOR, mechanistic target of rapamycin; NF-κB, nuclear factor kappa B; NLRP3, NOD-like receptor family pyrin domain containing 3; PD-L1, programmed death-ligand 1; PI3K, phosphoinositide 3-kinase; PKA, protein kinase A; PKC, protein kinase C; PLC, phospholipase C; ROS, reactive oxygen species; Wnt, wingless-related integration site; β-catenin, beta-catenin.
